# Probiotic Effects and Metabolic Products of *Enterococcus faecalis* LD33 with Respiration Capacity

**DOI:** 10.3390/foods11040606

**Published:** 2022-02-20

**Authors:** Yuehua Jiao, Han Yang, Nditange Shigwedha, Shuang Zhang, Fei Liu, Lanwei Zhang

**Affiliations:** 1 Key Laboratory of Dairy Science-Ministry of Education, Food College, Northeast Agricultural University, Harbin 150030, China; ava_1@126.com (Y.J.); y18166877720@163.com (H.Y.); szhang@neau.edu.cn (S.Z.); 2 Center of Drug Safety Evaluation, Heilongjiang University of Chinese Medicine, Harbin 150040, China; 3 School of Chemistry and Chemical Engineering, Harbin Institute of Technology, Harbin 150090, China; nditange@yahoo.com

**Keywords:** *Enterococcus faecalis*, respiration, probiotics, metabolic products

## Abstract

Respiration metabolism could improve the long-term survival of lactic acid bacteria (LAB); however, its effect on potential probiotic traits of LAB was not reported. The difference made by *Enterococcus faecalis* LD33 that was cultured under respiration-permissive and fermentation conditions, such as the biomass, metabolites, antimicrobial activity, tolerance to acid and bile salt, adhesion capabilities, and the ability to inhibit the proliferation of cancer cells were studied. Under a respiration-permissive condition, the final biomass of the culture was about twice as compared to that of fermentation condition. When the metabolites were measured, glucose was exhausted within 8 h. Two-folds of acetic acid, triple of both acetoin and diacetyl, and less than half of lactic acid, were accumulated under the respiratory-permissive condition. No discrimination of growth inhibition on *Salmonella enterica* serovar Typhimurium ATCC 14028 and *Shigella sonnei* ATCC 25931 was observed when *Enterococcus faecalis* LD33 was cultured under both conditions; however, under respiration-permissive condition, the strain presented significant antimicrobial activities to *Listeria monocytogenes* ATCC19111 and *Staphylococcus aureus* ATCC6538P. *Enterococcus faecalis* LD33 displayed relatively strong bile salt tolerance and adherence capability but weaker acid tolerance when undergoing respiration metabolism. There was no significant difference in the anti-cancer effect of the viable bacterial cells on both growth modes; however, the supernatant showed a higher inhibition effect on HT-29 cells than the live bacteria, and there was no significant difference between the supernatant and the 5-Fluorouracil (7 μg/mL). Consequently, the *Enterococcus faecalis* LD33 undergoing respiration metabolism could bring higher biomass, more flavor metabolites, and better antimicrobial and anti-cancer activities. This study extends our knowledge of respiratory metabolism in LAB and its impact on probiotic traits. *E. faecalis* LD33 qualifies as a suitable strain against foodborne pathogens, cancer therapy, and eventual application in the food and pharmaceutical industries.

## 1. Introduction

Lactic acid bacteria (LAB) are a phylogenetically diverse group named for their principal attribute in food fermentations, that is, the production of lactic acid [1]. Lactic acid finally inhibits the growth of LAB when accumulated in the culture [2,3]. LAB play an important role in various kinds of fermented food, mainly in dairy products (cheese and yogurt), fermented vegetables (sauerkraut, kimchi, and pickles), fermented cereals (sourdough), soy sauce, and meat products (sausages) [4,5,6,7]. LAB also contribute significantly to other fermentation processes, such as wine production [8].

A demonstration of altered metabolic end products, cytochrome formation, and hemin-dependent oxygen uptake indicated that the electron transport chain and cytochromes existed in hemin grown cultures [9,10]. LAB of several species, including *Lactococcus lactis*, *Enterococcus faecalis*, *Streptococcus* and *Lactobacillus* [10,11,12,13], are genetically equipped for aerobic respiration, providing them with a double metabolic life. They can switch from fermentation to aerobic respiration metabolism if equipped with exogenous heme (and menaquinones for some species, especially *Lactobacillus*) [14,15,16]. Respiration metabolism in *Lactococcus lactis* has been well documented, and it has a positive and significant impact on the bacterial biomass, O_2_ resistance, and long-term survival [10,17]. Consequently, the growth and survival of several strains of LAB can be dramatically improved under respiration-permissive conditions [18,19,20]. Furthermore, aerobic respiration positively impacts on the robustness and stress resistance of the strains, which can be beneficial for usage in food fermentation as probiotics and starter cultures [21,22,23].

Enterococci belong to LAB and are of importance in food fermentation; however, there are relatively few published reports about Enterococcal strains as probiotics compared to probiotic *Lactobacillus* strains. Certain Enterococci have been successfully used as probiotics to improve human or animal health. *E. faecium* SF68 [24,25,26] was the best known and probably the best researched probiotic *Enterococcus* strain in treating diarrhea and as an alternative to antibiotic treatment [27]. This strain showed the ability to inhibit the growth of *E. coli*, *Salmonella* serovars, *Shigella* spp. and *Enterobacter* spp., and equally could enhance the immune response to *Giardia intestinalis* in mice [28]. In vitro, it was shown to be resistant to low pH and tolerant to bile [29].

These findings opened up a new prospect on the LAB lifestyle, which must be understood to exploit their full potential [5,16]. Therefore, this study aimed to determine the differences of *E. faecalis* LD33 undergoing the lifestyle of fermentation and respiration at biomass, pH and fermentation metabolites in each period of the whole growth, at the inhibition effect of various indicator bacteria, at acid and bile tolerance, at the adhesion effect and inhibition effects of HT-29 human colon cancer cells. To our knowledge, this is the first comparison of the probiotic effects and the metabolites of the Enterococci when undergoing respiration and fermentation growth. In order to develop and use the benefits of *E. faecalis* LD33 brought by both metabolic styles, it was worthy of doing in-depth research on the strain that may be beneficial for future industrial exploitation when its safety is certified.

## 2. Materials and Methods

### 2.1. Strain and Growth Conditions

The strain LD33 used in this study was 1 of 18 strains isolated from the Inner Mongolia herders’ homemade cream. The strain was identified as *E. faecalis* by using the API 50 CH system (bioMérieux, Marcy l’Etoile, France) and analysis of 16S rDNA gene sequences. *E. faecalis* LD33 was cultured in an enriched M17 medium supplemented with 1% glucose (GM17). The strain was subcultured twice at 30 °C for 18 h before use. Hemin (Sigma-Aldrich, St. Louis, MO, USA) stock solution (0.5 mg/mL) was prepared (in 0.05 mol/L NaOH) and then pasteurized at 115 °C for 15 min. Twenty (20) μL of hemin stock solution was added to 1 mL of the medium. After inoculation with 3% (*v*/*v*) pre-cultures (routinely non-aerated cultures grown overnight in medium lacking hemin) in fresh GM17 medium with and without hemin respectively in Erlenmeyer flasks (filled to less than 1/10-volume capacity), growth experiments under the specified conditions were performed. Non-aerated cultures were grown without agitation and without hemin in the medium (fermentation conditions, FC), and aerated cultures were grown with shaking (250 rpm) and hemin (10 μg/mL) in the medium (respiration-permissive conditions, RPC). Aliquots of prepared cells were removed (at different times) for optical density (OD_600_) and pH measurements.

### 2.2. Metabolic Products Determination

Samples of 2 mL cultures were collected at appropriate harvesting time (1, 2, 3, 4, 5, 6, 7, 8, 9, 10, 11, 12, 14, 17, 21 and 24 h) after the start of growth under FC and RPC, and centrifuged at 9000× *g* for 5 min to remove the bacteria, and then the supernatants were stored at −20 °C until use. Before quantifying of glucose, lactate, acetate, acetoin and diacetyl, the supernatants were filtered through a 0.22 μm pore-size filter. The high-pressure liquid chromatography (HPLC 2695, Waters Corporation, Milford, MA, USA) was used for the determination as reported by Andersen et al. [30] and Starrenburg and Hugenholtz [31] with some modifications. Separation was performed with an Aminex HPX-87H column (300 × 7.8 mm, pre-packed HPLC carbohydrate analysis column, hydrogen form, 9 µm particle size, 8% cross-linkage, Bio-Rad, Richmond, CA, USA). H_2_SO_4_ (5 mmol/L) was used as the mobile phase at a0.5 mL/min flow rate. All products were detected by the Refractive Index Detector 2414 (Waters Corporation, Milford, MA, USA). The sample injection volume was 10 μL, the column temperature was 65 °C and the running time was 20 min. All experiments were conducted twice in three replicas.

### 2.3. Antibacterial Activity Assay

An agar well diffusion assay (AWDA) was used for testing the antibacterial activity of the cell-free supernatants (CFSs) of *E. faecalis* LD33 growth under FC and RPC, as described by Ammor, Tauveron, Dufour and Chevallier [32] and Thirabunyanon et al. [33] with some modifications. Oxford cups (6 mm in diameter) were placed on the surface of 1.5% (*w*/*v*) sterilized agar plates, and then 15 mL semi-solid Tryptic Soy Agar (TSA) medium (0.75%) containing 200 µL of the selected pathogenic strain (1.0 × 10^8^ CFU/mL) were overlaid to form a double-plate. Hundred (100) μL of CFS of the strain eliminated the effect of organic acid, and hydrogen peroxide was placed in each well, formed after Oxford cups were removed from the solidified medium. The plates were then incubated for 24 h at 30 °C and subsequently examined for inhibition zones. Inhibition was regarded as negative if no zone was observed around the wells. Each antibacterial activity was related to the diameter (mm) of the inhibition zone displayed. *Listeria monocytogenes* (*L. monocytogenes*) ATCC 19111, *Escherichia coli* (*E. coli*) ATCC 25922, *Staphylococcus aureus* (*S. aureus*) ATCC 6538P, *Salmonella enterica* serovar Typhimurium (*Salm.* Serovar Typhimurium) ATCC 14028, *Shigella sonnei* (*Sh. Sonnei*) ATCC 25931 were used as indicator pathogen strains, and all of them were cultured in TSA medium.

In order to eliminate the inhibition of organic acids in the CFSs, the test supernatants were adjusted to pH 6.5 with 4 mol/L NaOH and treated with catalase (1 mg/mL) to exclude the inhibition due to hydrogen peroxide production. The lactic acid of pH 6.5 was used as a control. Proteinase K, Pepsin, and trypsin (at a final concentration of 3 mg/mL) [34] were purchased from Sigma-Aldrich and used to validate the production of bacteriocin-like substances, respectively.

### 2.4. Survival in Acid and Bile Salt

To determine the tolerance of the strain under acidic conditions, the method of Watanabe et al. [35] was used with slight modifications. Overnight pre-cultures (10^9^ CFU/mL) were inoculated (1% *v*/*v*) in 25 mL of GM17 broth acidified to different pH (2.9, 2.6, 2.3 and 2.0) with 1.0 mol/L HCl, using unacidified broth as control. After incubation at 30 °C for 30 min under FC and RPC, respectively, the viability of the bacterial cells was determined by plating them on a GM17 agar medium and then incubated at 30 °C for 72 h. Acid tolerance of the strain was quantified from the number of viable cells, represented by log (N/N_0_), whereby N and N_0_ are the CFU of the acidified group and the control group, respectively.

The ability of a strain to grow in the presence of bile was determined according to the method of Succi et al. [36] with some modifications. The strain was inoculated (1% *v*/*v*) into GM17 broth with 0.3, 0.5 and 1% (*w*/*v*) of bile (Sigma-Aldrich). Cultures were incubated at 30 °C for 24 h under FC and RPC, respectively. The absorbency of each culture was measured at 600 nm, using bile salt-free broth as a control. The results were expressed as:

Bile tolerance (%) = [OD (sample)/OD (control)] × 100%

where, OD (sample) is OD_600_ of the culture containing bile salts; OD (control) is OD_600_ of the control culture without bile salts.

### 2.5. Adhesion Capability Assay

The adhesion of the strain to HT-29 cells was examined using an assay previously described by Chauvière et al. [37] and Zhang et al. [38] with a slight modification. Briefly, the HT-29 monolayers were prepared in six-well tissue plates, washed twice with phosphate-buffered saline (PBS) (Sigma-Aldrich). For each adhesion assay, the strains incubated under FC and RPC were collected by centrifugation and suspended in RPMI-1640 (Hycolone, Utah, UT, USA), were then added to each well to reach a ratio of 100:1 (10^8^ bacteria/10^6^ cell). After being incubated for 3 h at 37 °C in 5% CO_2_/95% air, the monolayers were washed 5 times with sterile PBS (37 °C) to remove un-adhered bacteria. Triton X-100 (0.1%), purchased from Sigma (Deisenhofen, Germany), was used to lyse the cells and release the adhered cells from the wall by incubated them at 37 °C for 30 min. The mixture was serially diluted, and the appropriate dilutions were plated onto M17 agar plates before being cultured at 37 °C for 48 h. The adherence index was expressed by CFU/mL for each mixture.

### 2.6. Inhibition of HT-29 Human Colon Cancer Cells

The 3-(4, 5-dimethylthiazol-2-yl)-2, 5-diphenyltetrazolium bromide (MTT) (Sigma-Aldrich) assay was carried out in 96 well tissue plates [33,39] to test the inhibition to human colon cancer cells HT-29 of the strain. Fifteen (15) μL of HT-29 cell suspensions (10^5^ cells/mL) and RPMI-1640 (120 μL for sample groups and 135 μL for the control group) were dispensed into each well, and the plates were incubated at 37 °C until almost all the cells were attached to the wall. The strains incubated under FC and RPC were centrifuged at 10,000 × g at 4 °C for 5 min. The pellet was washed three times with PBS and then suspended in RPMI-1640. Four sample groups were evaluated in this study, including 3 groups of different concentrations of live bacteria (10^5^, 10^6^ and 10^7^ CFU/mL suspended in 15 μL of RPMI-1640, separately) and a group of CFS (15 μL), and the samples were put into each well containing 135 μL RPMI-1640. Five-Fluorouracil (5-FU, 7 μg/mL as the final concentration) was used as a positive control. After incubation at 37 °C for 72 h in 5% CO_2_/95% air, 10 μL MTT solution (0.5 mg/mL in PBS) was added to each well and incubated for another 4 h to form the formazan precipitates, and then were solubilized by adding 150 μL DMSO. After 5 min incubation, the absorbance was measured at 490 nm by Eon’s microplate reader (BioTek, Winooski, VT, USA). Results were presented as the inhibition rates. All results were transformed into percentages based on their respective controls, calculated using the following equation:

Inhibition rate% = [1 − OD _(Sample)_/OD _(Control)_] ×100%

### 2.7. Statistical Analysis

The data obtained in this study were performed with SPSS 20.0 for Windows (SPSS Inc., Chicago, IL, USA) and expressed in means ± SD (n = 3 independent experiment). Statistical significance analysis was determined using one-way analysis of variance (ANOVA, SPSS 20.0), and then Duncan’s multiple range test was used for multiple comparisons. Values of *p* < 0.05 were considered statistically significant.

## 3. Results and Discussion

### 3.1. Respiration-Permissive Condition Leads to Biphasic Growth

**Figure 1:**
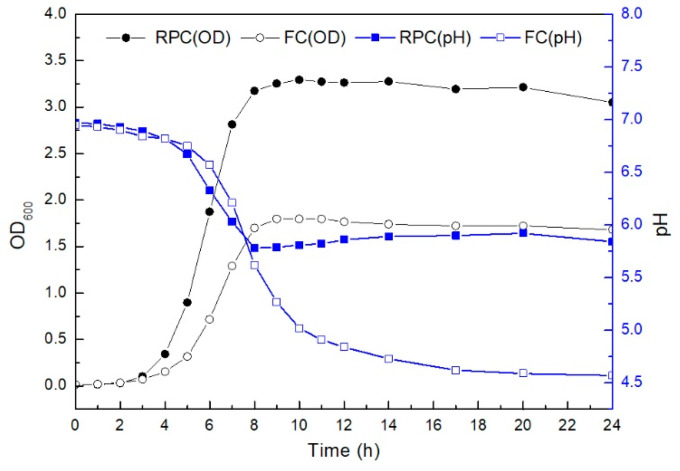
The differences in OD_600_ and pH for *E. faecalis* LD33 cultured under fermentative and respiration-permissive conditions respectively during the whole growth period (24 h), respectively. RPC and FC mean *E. faecalis* LD33 cultured under respiration-permissive and fermentation conditions, respectively.

The growth and pH of *E. faecalis* LD33 at appropriate time cultured under state of fermentation and respiration were compared, and the results are shown in Figure 1. The growth kinetics of *E. faecalis* LD33 cultured under both conditions were primarily similar during the first 3 h of growth. At 4 h (entering the exponential phase), the cell density of the respiratory-grown culture was significantly higher than that of the fermentation culture. During 4–8 h of growth, the initial pH of 6.9 dropped dramatically to 5.6 (for RPC) and 5.7 (for FC), respectively. *E. faecalis* LD33 culture entered stationary phase at the 8th h, the biomass of RPC culture was about twice as much as that of FC culture. At the same time, the pH value of RPC culture encountered a breakpoint and began a slightly upward to final pH 5.8. In contrast, the final pH of FC cultures was as low as 4.6.

The metabolic life of *E. faecalis* LD33 cultured under RPC was changed to the aerobic respiration metabolism at the very beginning of the exponential-growth phase period (at 4 h). The pH value could also verify that some changes in metabolic pathways were happening at this very time (begin from 4 h). Many experiments suggested that *L. lactis* can respire when oxygen and hemin are available [9,12,14]. In most of *L. lactis*, such as *L. lactis* MG1363 and *L. lactis* CHCC2862, the metabolism was altered just before the time when the cultures entered the stationary phase under fermentative conditions [9,40]. The metabolism of *Lb. plantarum* WCFS1 was changed at the exponential stage [13]. Contrary to those studies, the time of *E. faecalis* LD33 metabolism was altered at the very beginning of the early exponential phase other than just before entering the stationary phase of *L. lactis* MG1363 and *L. lactis* CHCC2862 or the exponential phase of *Lb. plantarum* WCFS1. Thus, the time of strains entering respiration from fermentation may differ among different strains, which may also be affected by the growth condition [9,22].

### 3.2. Metabolites Altered by the Respiration-Permissive Condition

**Figure 2:**
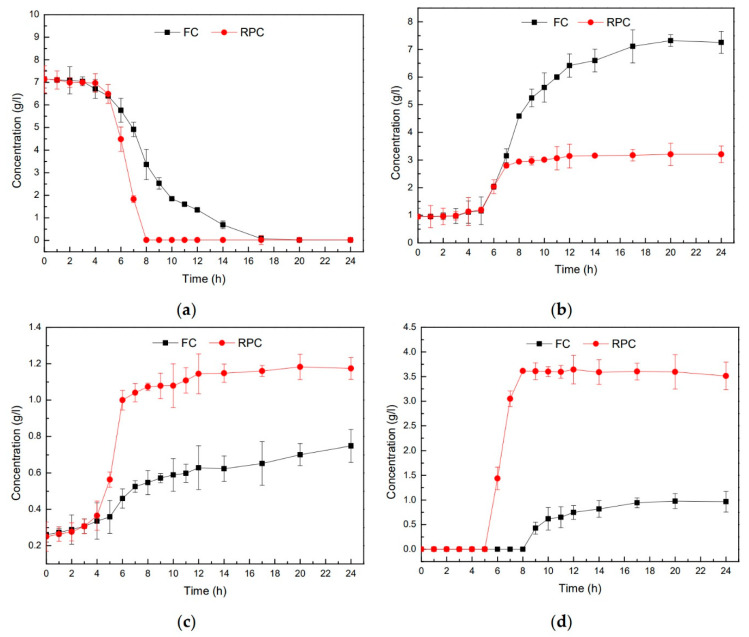
Metabolic production alterations of the strain *E. faecalis* LD33 cultured under FC and RPC. (**a**) Glucose, (**b**) lactate, (**c**) acetate, and (**d**) acetoin plus diacetyl, which all indicate the change of their concentration during the growth of 24 h. RPC and FC mean *E. faecalis* LD33 cultured under respiration-permissive and fermentation conditions, respectively.

Several metabolites were altered by respiration, including a considerable decrease in lactic acid accumulation and a substantial increase in acetoin and diacetyl accumulation. Both growth modes’ metabolites of *E. faecalis* LD33 were measured at specified time intervals. The contents of glucose, lactate, acetate, acetoin and diacetyl at different times are shown in Figure 2 (a, b, c, and d, respectively). Note that there were slight differences in the growth kinetics of *E. faecalis* LD33, especially between medium batches. In Figure 2, the content of all these four metabolites were basically the same in the supernatant under FC and RPC within the first five hours of growth. The differences could be identified at the 6th h, and because of the initiation of the exponential phase, a large amount of glucose was consumed. By the 8th h, glucose in the supernatant under RPC was exhausted. 

In contrast to supernatant glucose under FC, it was consumed less and more slowly until the 17th h. However, glucose was mainly utilized to produce lactic acid during the fermentation metabolism, and the final pH dropped to 4.6. In contrast, glucose was mainly utilized in the respiratory metabolism to produce acetic acid, and the final pH increased significantly to 5.8 (Figure 1 and Figure 2a,b).

Figure 2b showed that at 6 h, the lactic acid content of the supernatant under both culture conditions increased sharply. Two hours later, the lactic acid content of the supernatant under RPC reached the top, and the level remained unchanged for the rest of the whole period tested. In comparison, the lactic acid content of the supernatant under FC continued to accumulate as time passed. The trends of lactic acid production were the main factor affecting pH value, which caused it to continue to decline under FC and stop to decline until 8 h under RPC. In Figure 2c, higher acetate levels in RPC culture showed the most significant difference after 6 h of the growth, almost more than twice of that in FC culture; however, due to low acetic acid production, it contributed less to pH change compared to lactic acid.

Acetoin accumulates early during growth under RPC, and the amounts were clearly more than that in the other culture; the most incredible increment in acetoin occurred after 6 h of growth under RPC compared to that occurred after 8 h of growth under FC. These results can be seen in Figure 2d. Our results were in agreement with other previously reported in the literature. For example, Kaneko et al. [41] had reported that *Lactococcus lactis* subsp. *lactis* 3022 generated more biomass and transformed nearly all of the individual glucose substrate to both acetoin and diacetyl after being grown aerobically with hemin together with Cu^2+^. Duwat et al. [9] and Pedersen et al. [14] also reported that when respiration was activated by heme addition, lactic acid production was seen to diminish with an increase in the final pH. In addition, the yields of neutral metabolic byproducts of acetoin and diacetyl were massively increased. Interestingly, the production of all the indicated composites might work as a metabolic safety valve to redirect pyruvate [18]. Pyruvate conversion to acetoin accounted for around 20% of each glucose carbon exercised during respiration [14].

### 3.3. Antibacterial Effect of E. faecalis LD33 on the Pathogen during the Growth Modes

In this study, the supernatants were obtained after being cultured under either FC or RPC for 24 h each. AWDA was used to test whether the strain could produce bacteriocins against both Gram-positive and Gram-negative bacteria. The results were shown in Table 1. The inhibition effect of *E. faecalis* LD33 grown under RPC on *L. monocytogenes* and *S. aureus* was significantly higher than those grown under FC (*p* < 0.05). The comparison of the inhibition effect on *Salm. Typhimurium* and *Sh. Sonnei* between both FC and RPC culture showed no significant difference (*p* > 0.05). The CFS of the strain under both culture conditions could not inhibit the growth of *E. coli*.

The results suggested that inhibition effects of *E. faecalis* LD33 cultured under different conditions were different. The inhibition effect of the CFS (organic acid eliminated) on the indicator bacteria was somehow caused by the production of bacteriocin by the strain during the metabolic progress [32,42]. When the CFSs of the cultures were treated with protease K, pepsin and trypsin, the antibacterial activity for each was either decreased or abolished. Thus, we projected that the bacteriocin produced by *E. faecalis* LD33 might belong to Class Iia bacteriocins, which virtually partly did the antagonistic activity against the selected food pathogens, as their actions were not upset by low or neutral pH values. The literature revealed that Class Iia bacteriocins have strong antibacterial activities against *L. monocytogenes* (for example) and contain a conserved YGNGVXCXXXXCXV sequence motif at their N-terminus [43]. The typical bacteriocins of LAB initially possess a confined antibacterial range and inactivate nearly associated bacteria exclusively [44,45]; however, *E. faecalis* LD33 produces bacteriocin with a broader inhibitory spectrum and a more potent antimicrobial activity, which inhibited the growth of Gram-positive pathogenic and spoilage bacteria (such as *S.* *aureus* and *L. monocytogenes*) as well as some Gram-negative species (*Sh. Sonnei* and *Salm*. Typhimurium) under respiration mode especially. That can be because *E. faecalis* LD33 could produce more acetic acid, acetoin, and diacetyl. In addition, such changes in metabolic pathways brought in more energy to produce much more secondary metabolite (including bacteriocin) during aerobic respiratory metabolism than fermentation. Besides bacteriocins, acetic acid, acetoin and diacetyl have been part and parcel of antimicrobial compounds [46,47].

### 3.4. Tolerance of Acid and bile Salt and the Adherence Capability

**Figure 3:**
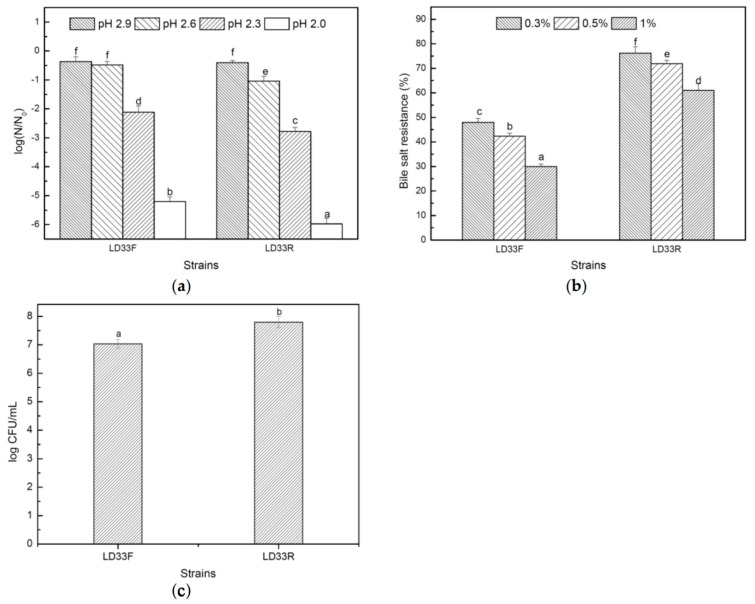
The probiotic effects of the strain *E. faecalis* LD33, including survival of *E. faecalis* LD33 (**a**) in an acid condition, (**b**) in bile salt condition, and (**c**) adhesion ability to colonic epithelium cells (HT-29 cells). LD33F and LD33R mean *E. faecalis* LD33 cultured under respiration-permissive and fermentation conditions, respectively. Different letters indicate significant differences (*p* < 0.05).

Acid and bile tolerances are considered as essential characteristics of LAB, enabling them to survive, grow and exert their probiotic functions in gastrointestinal transit [48,49,50]. The tolerance test results to acid and bile salt were shown in Figure 3a,b. The tolerance to low pH stress of *E. faecalis* LD33 was accounted for by the survival of cells of the strain treated 30 min in the GM17 broth of low pH (2.9, 2.6, 2.3 and 2.0) under FC and RPC. For both growth modes, the mortality of the strain increased as the pH decreased, though the cells that underwent fermentative mode were able to tolerant more acid stress. When the pH of the broth was 2.9, there was no significant difference between the acid resistance of the strain for both growth modes (*p* > 0.05). At pH 2.0, 2.3 and 2.6, the survival cells of the strain for fermentation growth were significantly higher than that of respiring growth (*p* < 0.05). When pH reduced to 2.0, a decrease of only 0.3 pH unit from pH 2.3 resulted in a drastic reduction in acid survival, translating to 5.2 and 6.0 in magnitude for FC and RPC, respectively. Interestingly, a drop of just 0.15 pH unit in both growth modes severely decreased acidic endurance.

The variations in acid endurance of the various growth modes at decreasing pH roughly corresponded with the level of radical synthesis and membrane integrity. The same findings as Watanabe et al. [35] were reported, respiring cells were more sensitive to acid exposure while fermentative cells were more acid-resistant. They performed flow cytometry experiments at decreasing pH to assess the radical formation and cell membrane integrity. Finally, they suggest that sudden death because of critical low pH is associated with the formation of radicals and loss of membrane integrity; however, hemin could activate the intracellular catalase activity of the strains undergoing respiration, which could reduce the damage of such oxidants to cells; likewise, to some extent, the respiratory condition is most prone to radical formation.

The results of bile salt tolerance are shown in Figure 3b. The growth of *E. faecalis* LD33 was inhibited by all three concentrations of bile salts, and at the same concentration *E. faecalis* LD33 of respiring growth appeared better tolerant to bile salts as compared to that of fermentative growth (*p* < 0.05). When the salt concentration was increased, the survival rate of the strain in both growth modes declined. The adhesion ability to colonic epithelium cells was also a selection criterion for probiotic strains [51]. The difference in the adhesion ability of *E. faecalis* LD33 for both growth modes was shown in Figure 3c. After 3 h incubation with HT-29, the adhesion ability of the strain for respiring growth was more than that for fermentative growth. The differences in bile salt tolerance and adhesion ability between any strain undergoing respiring and fermentative growth have not been reported before. Our results suggested many differences in the acid and bile salt tolerance and the adherence ability of *E. faecalis* LD33 for the two growth modes. The differences may be caused by the different metabolites of these two respective metabolism modes.

### 3.5. Growth Inhibition of HT-29 Human Colon Cancer Cells

**Figure 4:**
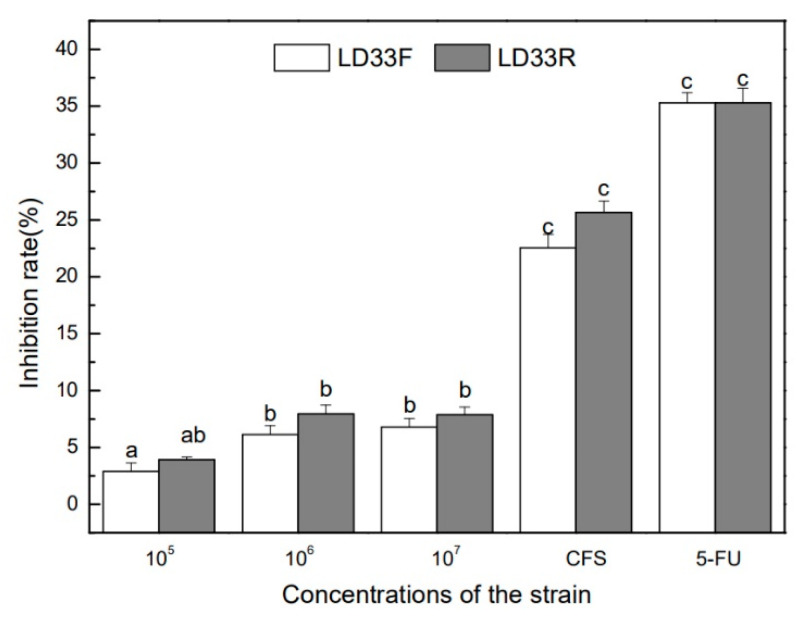
Inhibitory rates to HT-29 cells of *E. faecalis* LD33 cultured under FC and RPC. The four sample groups were different concentrations (1 × 10^5^, 1 × 10^6^ and 1 × 10^7^ CFU/mL) of *E. faecalis* LD33 in HT-29 cells (10^5^ cells /mL) suspension and the cell-free supernatant (CFS) of the strain. 5-FU (7 μg/mL) was used as a positive control. LD33F and LD33R mean *E. faecalis* LD33 cultured under respiration-permissive and fermentation conditions, respectively. Different letters indicate significant differences (*p* < 0.05).

The anti-cancer effects on the HT-29 cells of the live bacteria and the supernatant of *E. faecalis* LD33 in both growth modes via the MTT test revealed considerable variation. The anti-proliferative activity is shown in Figure 4. After *E. faecalis* LD33 in both growth modes was cultured for 24 h, the strains were co-cultured with the HT-29 colon cancer cells for 4 h. The results indicated that regardless of the live respiring cells and the live fermentative cells, or supernatant of the culture of both modes played an inhibition effect on the proliferation of HT-29. With the bacterial cells count increasing, its inhibition effect on HT-29 cells has also been enhanced. However, compared with the live bacterial cells, the supernatant’s inhibition effect was more significant (*p* < 0.05). 

Moreover, there was no significant difference (*p* > 0.05) in inhibition effect between supernatant and the positive control 5-Fluorouracil (5-FU, 7 μg/mL). The inhibition roles of all viable bacteria cells of *E. faecalis* LD33 (1 × 10^5^, 1 × 10^6^, and 1 × 10^7^ CFU/mL) under both growth modes on HT-29 cells were significantly fragilethan that of 5-FU (*p* < 0.05). Previous reports claimed that whole bacterial cells of lactobacilli had anti-proliferative activities against colon cancer cell lines HT-29, HCT-116 and SNU-C4 [51,52,53,54]. This result suggested that the supernatant showed a superior anti-cancer effect than different concentration live cells; however, there was no clear distinction between the two growth modes tested. It is feasible that the growth inhibition came from the more comprehensive inhibitory spectrum and strong antimicrobial activity of bacteriocin produced by *E. faecalis* LD33. Other researchers have also encountered that some bacteriocins (such as nisin, pediocin PA-1, microcin E492, enterocin heterodimer and divercin V41) had a selective cytotoxic effect against cancer cells [55,56]; however, that still needs further exploration for potential cytotoxicity mechanisms towards HT-29 cells exhibited by the bacteriocin produced by *E. faecalis* LD33.

## 4. Conclusions

The growth of *E. faecalis* LD33 undergoing respiration could bring unprecedented transformations of higher biomass and more flavor metabolites such as acetoin and diacetyl. The presumed bacteriocins and bacteriocin-like substances produced by *E. faecalis* LD33 had a broader inhibitory spectrum. Moreover, *E. faecalis* LD33 led a vigorous antimicrobial activity to *L. monocytogenes* and *S. aureus* and displayed a better performance to bile salt and adherence capability when underwent respiring growth. *E. faecalis* LD33 also had anti-proliferative activities against colon cancer cell lines HT-29. Its supernatant showed a superior anti-cancer effect than different concentrations of live cells, even if there was no significant difference between supernatant and the 7 μg/mL of 5-Fluorouracil. On the anti-cancer effect of *E. faecalis* LD33, there was no significant difference between the two growth modes. As a result, *E. faecalis* LD33 with respiration capacity can be a good strain for further research and applications in food, agriculture and pharmaceuticals. 

## Figures and Tables

**Table 1:** Antibacterial activity of the strain *E. faecalis* LD33 on the selected pathogenic bacteria.

	Diameter of the Inhibition Zone (mm)				
	*L. monocytogenes*	*S. aureus*	*E. coli*	*Salm.* Typhimurium	*S* *h. Sonnei*
LD33 R	18.26 ± 0.47 ^d^	16.58 ± 0.53 ^c^	_	14.21 ± 0.27 ^ab^	17.81 ± 0.49 ^d^
LD33 F	14.55 ± 0.25 ^ab^	15.53 ± 0.32 ^b^	_	15.01 ± 0.63 ^b^	18.57 ± 0.66 ^d^

All data were expressed as mean values ± SD (n ≥ 3); “_”(negative) means no zone was observed; The diameter (mm) of the inhibition zone contains the outside diameter of the Oxford cup (8 mm); Values followed by different letters are significantly different (*p* < 0.05); LD33R and LD33F mean *E. faecalis* LD33 cultured under respiration-permissive and fermentation conditions, respectively.

## Data Availability

The data presented in this study are available on request from the corresponding author.
